# Bone marrow infiltration in Langerhan’s cell histiocytosis - An unusual but important determinant for staging and treatment

**Published:** 2015-10-01

**Authors:** Mahendra Kumar, Man Updesh Singh Sachdeva, Shano Naseem, Jasmina Ahluwalia, Reena Das, Neelam Varma, R K Marwaha

**Affiliations:** 1 Assistant Professor, Department of Pathology, Institute of Medical Sciences, Banaras Hindu University, Uttar Pradesh, India; 2 Associate Professor, Department of Hematology, Postgraduate Institute of Medical Education and Research, Chandigarh, India; 3 Assistant Professor, Department of Hematology, Postgraduate Institute of Medical Education and Research, Chandigarh, India; 4 Additional Professor, Department of Hematology, Postgraduate Institute of Medical Education and Research, Chandigarh, India; 5 Professor, Department of Hematology, Postgraduate Institute of Medical Education and Research, Chandigarh, India; 6 Professor and Head, Department of Hematology, Postgraduate Institute of Medical Education and Research, Chandigarh, India; 7 Professor, Department of Pediatrics (Hematology-Oncology Unit), Postgraduate Institute of Medical Education and Research, Chandigarh, India

**Keywords:** Bone marrow, Histiocytes, Langerhan’s cells

## Abstract

**Background:** Langerhans' cell histiocytosis (LCH) is a reactive proliferative disease of unknown pathogenesis characterized by proliferation of Langerhans cells. Involvement of bone marrow (BM), liver and lung are related to high risk factors and poor survival. The aim of this report is to highlight the clinical and haematological findings of 5 cases of LCH with BM infiltration which may help to predict involvement of BM.

**Case series:** Five cases of Langerhan’s cell histiocytosis with bone marrow infiltration were retrieved from archives of Department of Hematology, PGIMER and Chandigarh for review and further analysis. Male to female ratio was 3:2 with mean age of 9.4 months. Two out of 5 patients had obvious skull swelling; however, radiography of the skull revealed lytic lesion of skull in 4 cases and 2 had skin rashes. Hepatomegaly was present in 4 cases and 2 of whom also had lymphadenopathy and splenomegaly. All patients had anaemia at the time of presentation. Bone marrow aspiration and trephine biopsy in all 5 cases revealed infiltration by large histiocytes with abundant cytoplasm and coffee bean shaped nucleus. Nodules of these Langerhans cells with admixture of eosinophils were seen on trephine biopsy. Immunohistochemistry showed positivity for CD1a stain.

**Conclusion:** BM evaluation is important in LCH patients to categorize disease which further determines the type of therapy to be given. Clinical details may help to predict the BM involvement; however, demonstration of CD1a positive cells in marrow is most important tool to diagnose marrow infiltration by LCH.

## Introduction

 Langerhans’ cell histiocytosis (LCH) is a rare proliferative disorder of unknown etiology that occurs more frequently in children than in adults. Until now, this enigmatic disease has been variously classified as a neoplastic process, a reactive disorder, or an aberrant immune response. Recently, biologic studies have supported the contention that LCH represents a clonal proliferative disorder of cells closely related to Langerhans’ cells (LCs).^   1^ The disease ranges in severity from a curable solitary lytic bone (more frequently in adults) to a fatal leukemia-like disorder (primarily affecting infants). Intermediate forms show a variable course which is characterized by bony and skin lesions and with or without organ dysfunction. The diagnostic criteria for Langerhans' cell histiocytosis have been defined in a workshop of the Histiocyte society.^   2^ We present here five cases diagnosed as Langerhans' cell histiocytosis in infancy with involvement of bone marrow (BM).

## CASE SERIES

 Retrospective analysis of BM examination reports from Department of Hematology, PGIMER, Chandigarh, was carried out between the time periods of January 2007 to July 2011. A total of 19 patients with LCH were retrieved and bone marrow infiltration was detected by LCs in 5 patients. Clinico-hematological profiles of these 5 patients are discussed here.

Among 5 patients of LCH with BM infiltration, diagnosis of LCH was confirmed by histopathological examination of skin biopsy (n=2), lymph node excision biopsy (n=1) and fine- needle aspiration cytology of skin nodules (n=2). Clinico-hematological profiles of all 5 cases are summarized in Table-1. Mean age was 9.2 months with M: F of 3:2. The most common presentations were fever and hepatomegaly. Bone lesions were seen in all 5 cases and skull involvement was seen in 4 cases. Anemia was present in all cases, ranging from mild to severe. Total leukocyte count (TLC) was within normal limit in all cases except one who showed leukocytosis. Three patients had normal platelet counts, and one patient had thrombocytopenia and another one had thrombocytosis. None of the above-mentioned patients showed central nervous system (CNS) or lung involvement; however, one patient had ear involvement (Table 1).

**Figure 1:**
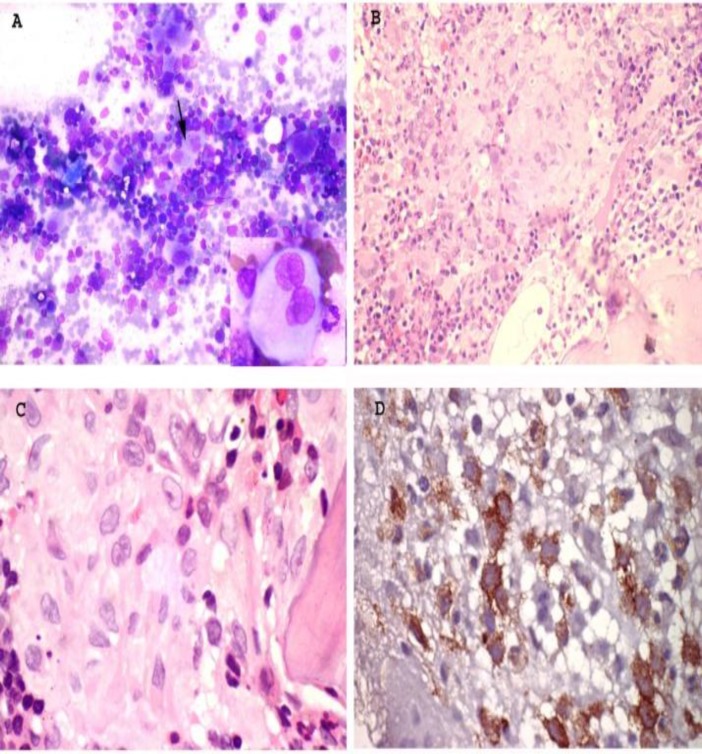
Panel of microphotographs from bone marrow aspirate and trephine biopsy of a case of LCH indicating BM infiltration by Langerhans Cells: **(A)** Bone marrow aspirate showing cellular marrow with scattered large histiocytes having lobated nuclei and abundant cytoplasm (arrow) (Giemsa stain, magnification 20x) **(B)** Bone marrow trephine biopsy showing a small nodule comprising of Langerhan cells bordered by eosinophils (Hematoxylin and Eosin stain, magnification 10x) **(C)** Bone marrow trephine biopsy showing the morphology of Langerhans cells at higher magnification, the cells having abundant cytoplasm and grooved nuclei (Hematoxylin and Eosin stain, magnification 40x) **(D)** Immunohistochemistry for CD1a showing cytoplasmic positivity in Langerhans cells (magnification 40x).

Bone marrow aspiration and trephine biopsy revealed normocellular marrow in 4 patients, whereas 1 patient had a hypocellular bone marrow. In addition to the increased interstitial histiocytes in all patients, 2 showed sheets and 1 showed cluster of histiocytes. Eosinophils were increased in all cases; however significant hemophagocytosis was noticed in only 1 case. CD1a immunohistochemical stain clearly highlighted the infiltrating Langerhans cells (Figure 1D).

## Discussion

 The incidence of LCH ranges from 2.6 to 6 per million in different study groups.^   3^ There are three main clinical subtypes that are encompassed by the term LCH.^   4^ The first variant is a unifocal and commonly involves bone (up to 80% of cases), lymph nodes, or lungs as primary targets.^4^ The second subtype is considered to be multifocal and involves several sites in one organ system (single system, multiple sites).^   4^ The third subtype affects multiple sites in multiple organ systems, and is most prevalent in young children and infants.

Recent studies have defined clinical categories depending on the extent and localisation of the disease at the time of evaluation: Single System LCH (SS-LCH) and Multi system LCH (MS-LCH). SS-LCH is defined as one organ/system involved (uni or multifocal); however, MS-LCH is defined as two or more organs/systems involved with or without involvement of “Risk Organs”. Risk Organ includes liver, lung, spleen and BM. This stratification of disease is important as it determines the mode of therapy, prognosis and survival of patients. ^5^^,^^6^

Most common organ involved is bone followed by skin. Involvement of lung, liver, lymph node and soft tissue is less common ^3^^,^^7^^,^^8^ but involvement of BM is very unusual. ^9^^–^^12^ The present study reports 5 cases of LCH with involvement of BM (26.3%) out of 19 diagnosed cases of LCH. Another study has shown around 33.3% of BM involvement,^   12^ however, larger studies have shown relatively lesser number of involvement, ranging 2 to 7.5%. ^9^^,^^10^ All 5 patients were infants with median age of 9.2 months and M: F ratio 3:2. It supports other studies that multisystem involvement usually occurs in infants^   4^ and more common in males presenting poor prognosis.^   10^

BM examination is an important investigation to categorize the disease and secondly, to determine the mode of treatment. ^7^^,^^9^^,^^10^ BM involvement is usually associated with multisystem LCH^   13^ and associated with poor prognosis. ^9^^–^^11^ In the present study, BM examination changed the category of only 1 case from single system to multi system; however, other 4 cases were already in multisystem category before marrow assessment. In our study, bony lesion, hepatomegaly and fever were most common presentations (80%) and we may conclude that these features are more often associated with marrow involvement.

**Table 1:** Cinico-hematological profile of LCH patients with BM infiltration

	**Case 1**	**Case 2**	**Case 3**	**Case 4**	**Case 5**
**Features** **Age (months)**	11	7	5	11	12
**Sex**	M	M	M	F	F
**Fever**	+	+	-	+	+
**Skin rash**	+	-	-	+	-
**Hepatomegaly**	+	+	+	-	+
**Splenomegaly**	+	-	-	-	+
**Lymphadenopathy**	+ (Cervical)	-	-	-	+ (Generalized)
**Bony lesion**	+ (skull)	+ (skull)	+ (skull)	+ (leg) Developed after BM staging	+ (skull)
**Haematological profile** ** Hb (gm/dl)** ** TLC (cell/L)** ** Platelets (/L)**	9.6 6.5 × 10^9^ 430 × 10^9^	5.4 9.7 × 10^9^ 693 × 10^9^	4.2 6.6 × 10^9^ 293 × 10^9^	10.5 14.7 × 10^9^ 459 × 10^9^	8.3 4.6 × 10^9^ 100 × 10^9^
**Soft tissue**	-	+ (scalp )	+ (scalp)	+ (leg)	-
**Other site/CNS/Lung**	-	-	-	Ear (Otitis media)	-
**Bone marrow findings**					
**Cellularity**	Normocellular	Normocellular	Normocellular	Mildly hypocellular	Normocellular
**Histiocytes**	Scattered and sheets	Scattered	Scattered	Scattered and sheets	Scattered and clusters
**Hemophagocytosis**	Present	Not seen	Not seen	Not seen	Not seen
**Eosinophils**	Increased	Increased	Increased	Increased	Increased

Two patients had skin rash, splenomegaly and lymphadenopathy. Regarding haematological parameters, all patients were anaemic and only 1 patient had thrombocytopenia, however, none had neutropenia or leucopenia. In the present study, none of the patients qualified criteria for haematological dysfunction ^5^^,^^6^  even after marrow involvement. Some studies have demonstrated hematological dysfunction without involvement of BM. ^9^^,^^10^  This paradox may lie in earlier studies ^9^^,^^10^  following modified Lahey's criteria^   14 ^ for organ dysfunction; however, we followed recent recommendations for organ dysfunction. ^5^^,^^6^  If we follow modified Lahey’s criteria, then 4 of our patients will come in a hematological dysfunction category. So, modified Lahey’s criteria seem to be superior in predicting organ dysfunction and indirectly indicating marrow involvement.

The present study may suggest that BM evaluation should be performed in patients even with single cytopenia which is slightly different from recent recommendations, saying bicytopenia is needed to define hematological dysfunction.^   6 ^

In all patients, marrow cellularity was normocellular to mildly hypocellular which is well correlated with the study by Minkov M. et al.^   15 ^ There were plenty of scattered as well as clusters of histiocytes in BM of all 5 cases, having coffee bean nuclei and abundant pale basophilic cytoplasm. Minkov M. et al. showed that there were no significant correlations between number of histiocytes and single or multisystem LCH.^   15 ^ Only 1 case showed significant hemophagocytosis. So, hemophagocytosis does not correlate to marrow involvement which was also demonstrated by Minkov. et al.^   15 ^ We demonstrated plenty of CD1a positive cells in all cases, which was well correlated with marrow infiltration. ^7^^,^^15^  Immunohistochemistry for langerin can also be done as a marker for Langerhans cells. All cases showed increased eosinophils in bone marrow and 1 case showed bone marrow fibrosis.

In our report, Vinblastin (VBL) and Prednisolone were given to all patients for 6 weeks. Four patients showed better responses (i.e. regression of signs and symptoms with no new lesion) and 1 showed intermediate response (i.e. persistence of signs and symptoms but no new lesion). Second course of same drugs was given for 6 weeks, followed by maintenance therapy (VBL + Prednisolone + Mercaptopurine). The maximum duration of chemotherapy was extended to 12 months and none of the patients showed relapse or progression of disease until the last follow-up.

## CONCLUSION

 The study demonstrated that clinical profile of LCH patient could be an important clue to predict BM infiltration as it is more frequently observed in infant with multisystem disease. Bone marrow involvement is suggested by the presence of aggregates of histiocytes with the characteristic morphology of Langerhans cells along with increased eosinophils. Meanwhile, demonstration of CD1a positive cells on immunohistochemistry is an important diagnostic tool.
